# Development of Osteopenia During Distal Radius Fracture Recovery

**DOI:** 10.1016/j.jhsg.2022.09.001

**Published:** 2022-09-27

**Authors:** Imran S. Yousaf, Gianna M. Guarino, Kavya K. Sanghavi, Tamara D. Rozental, Kenneth R. Means, Aviram M. Giladi

**Affiliations:** ∗ The Curtis National Hand Center, MedStar Union Memorial Hospital, Baltimore, MD; † MedStar Health Research Institute, Hyattsville, MD; ‡ Division of Hand and Upper Extremity Surgery, Department of Orthopedics, Beth Israel Deaconess Medical Center, Harvard Medical School, Boston, MA

**Keywords:** Bone mineral density loss, Distal radius fractures, Disuse osteopenia, Orthopedic injuries, Osteoporosis

## Abstract

**Purpose:**

To determine the degree of disuse osteopenia (DO) and factors associated with its development during treatment of distal radius fractures (DRFs).

**Methods:**

We retrospectively reviewed charts and radiographs of patients with DRFs treated with and without surgery at 2 health care systems. We defined DO as a >10% drop from initial to 6-week second metacarpal cortical percentage and 6-week absolute second metacarpal cortical percentage <60%. Bivariate analyses were performed to evaluate associations between treatment type, patient and fracture characteristics, and radiographic measurements with odds of developing DO. Significant associations were included in multivariable analyses, adjusting for patient and fracture characteristics.

**Results:**

Approximately 18% of 517 included patients met the criteria for development of DO (n = 93). Bivariate analysis showed that surgical treatment was associated with lower odds of developing DO, whereas advancing age was associated with increased odds. In adjusted multivariable models, only advancing age was associated with increased odds of developing DO.

**Conclusions:**

A fairly important proportion of patients with DRF develop hand DO 6 weeks after surgical or nonsurgical treatment. The clinical relevance of this finding is uncertain and requires further investigation.

**Type of study/level of evidence:**

Prognostic IV.

Distal radius fractures (DRFs) are the second most common orthopedic injuries in the elderly. They can be managed surgically, most often with open reduction internal fixation (ORIF), or nonsurgically.^1^ Surgical and nonsurgical treatments generally have different periods of immobilization or limited limb use. Bone mineral density (BMD) decreases in disused limbs, known as disuse osteopenia (DO).^2^ Loss of BMD at the DRF site can occur with nonsurgical treatment and in the ipsilateral and contralateral hand after distal forearm fracture.^3^^,^^4^ Dual-energy x-ray absorptiometry (DEXA) shows that systemic decreases in BMD can also occur with reduced weight-bearing activities.^5^^,^^6^ Although lower systemic BMD has not been found to predict DRF outcomes,^7^^,^^8^ it can impact fracture stability during healing.^9^ The impact of DRF treatment type on the development of DO has not been clearly established.

Osteoporosis is commonly identified using DEXA. However, in 2018, Schreiber et al^10^ reported a technique using the second metacarpal cortical percentage (2MCP) measured on standard hand radiographs as an indicator of global osteoporosis/osteopenia and validated it using DEXA. Our study had 2 aims: (1) to use 2MCP to quantitatively determine if DO occurs during the nonsurgical and surgical treatment of DRF, and (2) to determine factors associated with developing DO after DRF. We hypothesized that specific patient and fracture characteristics would increase the odds of developing DO after DRF and that ORIF would be associated with reduced development of DO compared with nonsurgical treatment.

## Materials and Methods

### Study population

**Figure 1:**
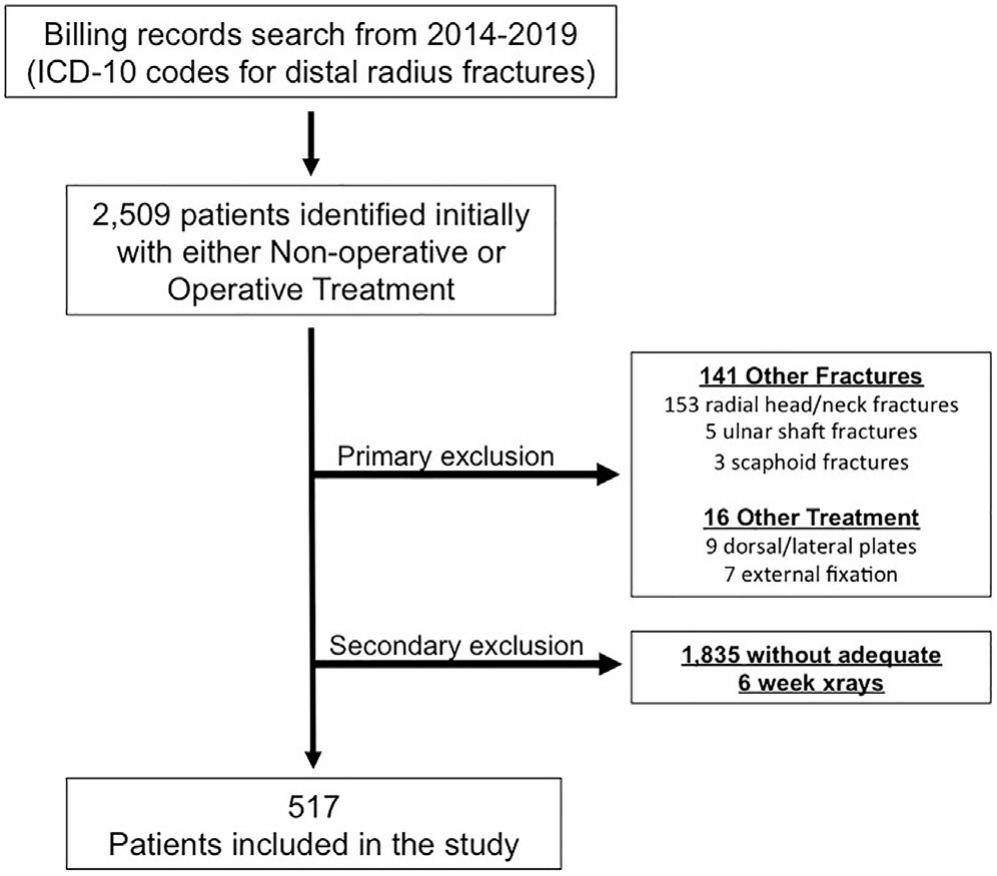
Flowchart of cohort identification.

Following institutional review board approval at each site, we reviewed medical records for patients treated at 2 health care systems from January 2014 to May 2019 with a diagnosis of DRF. We evaluated all patients with (1) DRF treated with immobilization or with ORIF with a volar locking plate and (2) digitally stored wrist radiographs, including posteroanterior and lateral views at the time of injury and a minimum of 6 weeks after injury or surgery. Many providers did not routinely obtain follow-up radiographs beyond 2 weeks. Additionally, both locations changed radiology programs during the study period. Patients with previous images that could not be measured consistently within the same program were excluded. Patients who were less than 18 years old, had additional fractures other than ulnar styloid fractures, had a prior fracture in the same wrist, had dorsal or lateral plate ORIF, had external fixation, or had closed reduction without available postreduction radiographs were also excluded (Fig. 1). We excluded the relatively rare dorsal or lateral plate ORIF for methodologic consistency to avoid any variability because of surgical approach or plate stability/shielding.

All included patients who underwent nonsurgical treatment were immobilized for a total of 5 weeks or slightly more. All included patients who underwent ORIF were immobilized for approximately 2 weeks and began range of motion. Patients deviating from this protocol by >1 week were excluded.

### Radiographic measurements

Using our institution’s digital imaging software (Centricity PACS), we made 5 primary measurements on radiographs using previously described techniques^11^^,^^12^: volar tilt, radial inclination, radial height, ulnar variance, and intra-articular displacement. Measurements were made by 2 trained research assistants and compared; discrepancies were evaluated by a fellowship-trained hand surgeon who made final measurement determinations. Depending on whether the patient was treated without or with surgery, these measurements were made on injury, postreduction, intraoperative, and final follow-up radiographs. If intraoperative radiographs were not present, other available images were used, provided they were taken <2 weeks after surgery. We also recorded DRF comminution, number of intra-articular fracture lines, and ulnar styloid fractures when present.

### Primary outcome

**Figure 2:**
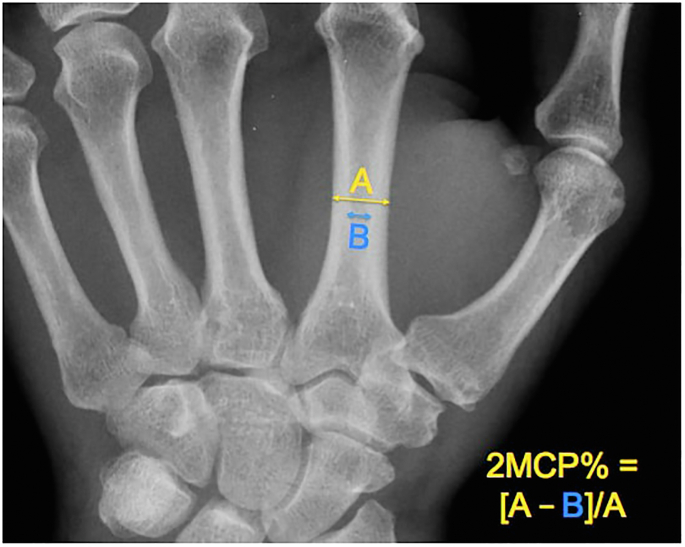
Ratio of cortical diameter relative to total diameter to calculate 2MCP as a measure of global BMD.

The 2MCP measured on initial injury radiographs was used as an indicator of global BMD. This was calculated on a true posteroanterior view that included enough of the hand to allow for the measurement.^10^ The calculation was made by taking the difference between the total and cortical diameter of the second metacarpal and dividing it by its total diameter (Fig. 2). Values were categorized as >60% or ≤60%. We defined the development of DO as (1) a >10% drop from initial to final (∼6 weeks) 2MCP and (2) when final 2MCP was ≤60%.^10^ The 60% cutoff was chosen as it was shown to optimize sensitivity (88%) and specificity (60%) for identifying patients with osteopenia, defined as DXA T score between −1 and −2.5 at the hip.^10^ Additionally, although studies have shown varying sensitivities for discerning osteopenia from healthy patients, the 60% cutoff for osteopenia was preferred because of its significantly high interobserver reliability,^10^ not evaluated in other reports.^13^ In addition, the 10% drop was used to define DO based on prior studies showing 9%–13% bone loss in the forearm after femoral fractures^14^^,^^15^ and up to 8% bone loss in DRFs.^16^

### Patient and fracture characteristics

Covariates included age, sex, race/ethnicity, fracture stability, fracture type, body mass index, and Charlson Comorbidity Index (CCI) scores. Age was used as a continuous measure. Race/ethnicity was categorized as White, African American, Hispanic, and Other. A fracture was considered unstable per LaFontaine criteria if on the initial x-ray there were 3 or more of the following criteria present^17^: dorsal tilt >20°, dorsal comminution, intra-articular fracture, ulnar styloid fracture, and age >60 years. A dichotomous variable was created accordingly for stable or unstable fractures. Fractures were also categorized according to the AO/OTA classification system.^18^ Body mass index was divided into 4 categories: underweight (<18.5), normal (18.5–24.9), overweight (25.0–29.9), and obese (>30.0).^19^ The CCI score was calculated for each patient and categorized based on the degree of comorbidity burden as none (0), medium (1 or 2), or high (>3).^20^^,^^21^ We also explored the inclusion of DEXA results and had insufficient data available.

### Statistical analysis

Patient demographics and fracture characteristics were summarized as appropriate depending on distribution patterns. Variance inflation factors were assessed to ensure that the primary covariates had low multicollinearity. Bivariate linear regression models were used to evaluate associations between patient or fracture characteristics and odds of developing DO. Covariates with *P <*.1 on bivariate analysis were included in a multivariable model. The statistical significance level was set at.05 for all other analyses. All analyses were conducted in Stata 15 (StataCorp).

## Results

A total of 517 patients were included (Fig. 1, Table 1). The median age was 58.9 years (standard deviation, 17.3 years), with 75.9% of patients being female and 69.1% being White. Most patients had nonsurgical treatment. More than 67% of patients had generalized osteopenia at baseline. Approximately 18% of all patients met our criteria for the development of DO during their DRF management (93 patients, 17.9%). A mean decrease of 9% from initial to final 2MCP was observed in patients with DO, compared with a mean decrease from initial to final 2MCP in the overall group of 0.2% (Table 1).

**Table 1:** Summary of Descriptive Statistics

Characteristics	All Patients (N = 517) N (%), or Median (IQR)	Without DO (N = 424) N (%), or Median (IQR)	With DO (N = 93) N (%), or Median (IQR)	*P* value∗
Age (years)	58.9 (47.9–68.6)	57.8 (46.6–68.2)	61.5 (54.6–74.3)	**.04**∗,†
Sex				.52
Male	124 (24.0)	104 (24.5)	20 (21.5)	
Female	392 (75.9)	319 (75.4)	73 (78.49)	
Race				.97
White	356 (69.1)	292 (69.19)	64 (68.82)	
Black	110 (21.3)	89 (21.09)	21 (22.58)	
Asian	26 (5.05)	22 (5.21)	4 (4.30)	
Other	23 (4.47)	19 (4.50)	4 (4.30)	
Body mass index				.96
Normal	178 (34.4)	148 (34.9)	30 (32.2)	
Underweight	23 (4.4)	19 (4.4)	4 (4.3)	
Overweight	163 (31.5)	133 (31.37)	30 (32.2)	
Obese	153 (29.5)	124 (29.25)	29 (31.1)	
CCI category				.16
No comorbidity	121 (23.4)	106 (25)	15 (16.1)	
Medium comorbidity	209 (40.4)	170 (40.0)	39 (41.9)	
High comorbidity	187 (36.1)	148 (34.9)	39 (41.9)	
LaFontaine criteria				.37
Stable	227 (43.9)	190 (44.8)	37 (39.7)	
Unstable	290 (56.0)	234 (55.1)	56 (60.2)	
Surgical status				
Nonsurgical	303 (58.6)	239 (78.8)	64 (21.1)	Reference
Surgical	214 (41.3)	185 (86.4)	29 (13.5)	**.03**∗,†
AO/OTA classification				.91
A type	165 (31.9)	134 (31.60)	31 (33.33)	
A1	8 (1.5)	7 (1.65)	1 (1.08)	
A2	101 (15.6)	80 (18.87)	21 (22.58)	
A3	56 (10.8)	47 (11.08)	9 (9.68)	
B Type	77 (14.8)	59 (13.92)	15(16.13)	
B1	57 (11.0)	44 (10.38)	13 (13.98)	
B2	11 (2.1)	9 (2.12)	2 (2.15)	
B3	8 (1.7)	8 (1.89)	0 (0)	
C Type	276 (53.3)	229 (54.01)	47 (50.54)	
C1	97 (18.7)	79 (18.63)	18 (19.35)	
C2	94 (18.1)	79 (18.63)	15 (16.13)	
C3	85 (16.4)	71 (16.75)	14 (15.05)	
Initial 2MCP	54.7 (47.7–63.4)	55 (47.6–64.1)	53.7 (47.8–59.5)	.36
Final 2MCP	54.5 (46.2–61.8)	56.7 (48.7–63.4)	44.7 (39.8–50.6)	**<.001**∗,†

AO/OTA = AO Foundation/Orthopaedic Trauma Association

∗ *P* values for chi-square test or median test for characteristics between patients with and without disuse osteopenia.

† Bolded values are statistically significant.

On bivariate analysis, surgical DRF treatment was associated with lower odds of developing DO (0.58 [95% confidence interval, 0.36–0.94]; *P <*.10). Increasing age was associated with increased odds of developing DO (1.02 [95% confidence interval, 1.00–1.03]; *P <*.10). There were no significant associations between the development of DO and initial or final radiographic parameters, sex, age >60 years, race, unstable fractures, AO/OTA classification, CCI score, or body mass index (Appendices A and B, available on the Journal’s website at www.jhsgo.org).

After including the treatment method and age in multivariable analysis, only age was still associated with increased odds of developing DO (1.02 [95% confidence interval, 1.00–1.05]; *P <*.05) (Table 2). All variance inflation factors were <3, indicating low multicollinearity (Appendix C, available on the Journal’s website at www.jhsgo.org).

**Table 2:** Adjusted β Estimates for Multivariable Linear Regression Model with Disuse Osteopenia as Outcome Measure

Characteristics	β Estimates (95% CI)	*P* Value
Surgical status		
Nonsurgical	Reference	
Surgical	0.64 (0.39–1.06)	.09
Age (years)	1.02 (1.00–1.05)	**.02**∗
CCI category		
Low comorbidity	Reference	
Medium comorbidity	0.82 (0.36–1.86)	.65
High comorbidity	0.59 (0.20–1.74)	.34

∗ Bolded values are statistically significant.

## Discussion

In more than 500 patients with DRF, our definition of DO occurred in ∼18% of patients regardless of treatment type. However, across all analyses, increasing age was the only factor significantly associated with developing DO, with unclear clinical significance considering the overall low effect. Although significantly associated with lower odds of DO in preliminary analyses, surgical treatment and abnormal and mean changes in certain radiographic parameters were not significant in multivariable models. Ultimately, aside from age, none of the covariates we evaluated were significantly associated with DO, even though a substantial portion of our cohort developed DO regardless of whether they had generalized osteopenia at baseline.

Earnshaw et al^22^ prospectively evaluated BMD loss via DEXA in a total of 107 postmenopausal patients undergoing nonsurgical DRF treatment. While half underwent closed reduction and 97% received immobilization in a plaster cast, more than 51% of patients developed systemic and local osteopenia, most in the hip, followed by the radius, and least in the spine. However, a quarter of their patients also had a history of wrist fracture, which could have accelerated BMD loss or indicated lower BMD at baseline.^23^^,^^24^ Bone mineral density measurements were recorded within 2 weeks of the fracture, whereas ours had a minimum of 6 weeks before measurement. Although osteopenia developing in the short-term has been reported,^25^^,^^26^ others have observed osteopenia peaking 6 weeks after injury and lingering for months to years after treatment.^16^ Although it remains unclear at what particular period osteopenia develops during DRF treatment, it is clear that BMD loss does occur for a substantial number, perhaps because of immobilization, systemic inflammation, and hormones controlling calcium homeostasis.^24^

Earnshaw et al^26^ also found that a slightly greater proportion of patients with DRF >66 years old had osteopenia at the distal radius. While older age is naturally associated with developing osteopenia, others report significant BMD loss even in adolescents during DRF cast-immobilization. This underscores the potential for BMD loss with the immobilization and metabolic factors associated with acute fractures.^27^, ^28^, ^29^, ^30^, ^31^ In our study, advancing age was associated with a slightly increased odds of developing DO during DRF care. A potential explanation is that fractures occurring in younger patients may indicate pathologically low baseline BMD. In contrast, with advancing age, BMD loss may be more likely to develop following an acute fracture.^22^

In a prospective longitudinal cohort of 40 patients with DRF managed nonsurgically with 6 weeks of immobilization and compared with age-matched controls, Ingle et al^16^ observed greater BMD loss via DEXA occurring within 1 year of injury in 35% of patients. Total BMD of the fractured hand significantly decreased by 6.1% compared with that in nonfractured controls. We observed a 9% decrease in 2MCP in those with DO and at a much shorter interval; however, we cannot reliably compare changes in DEXA BMD with changes in 2MCP. Future investigators should consider the potential impact of baseline or fracture-induced sedentary behavior on BMD changes following DRF.^32^^,^^33^

The authors of another report prospectively assessed BMD changes in 18 postmenopausal patients undergoing nonsurgical DRF treatment.^3^ Using high-resolution peripheral quantitative computerized tomography, they observed differential BMD loss occurring in all patients 1-4 weeks after DRF. Despite increases in the mean total and trabecular bone density, the mean cortical BMD decreased substantially. Ultimately, 50% of the patients met DEXA criteria for osteopenia. We similarly observed a significant decrease in final 2MCP by an average of 9% from initial values in the injured limb in our patient population.

Surgical fixation of fractures may lead to local bone atrophy by impairing blood flow, as seen in animal models.^34^^,^^35^ Stress shielding and impaired periosteal circulation also drive local bone loss, as confirmed in diaphyseal forearm fractures treated with ORIF.^36^^,^^37^ However, bone loss in the hand after DRF treated with volar plates has not been widely reported. We hypothesized that ORIF would be associated with less DO, perhaps because of the advantage of early mobilization.^38^^,^^39^ Although not statistically significant after controlling for age and comorbidities, given that on bivariate analysis, the percentage of patients who developed DO was lower for the surgical group and could be clinically relevant, we may have been underpowered to confirm it as an independent factor. Future studies with prospective protocols can quantify the impact of mobilization and its timing on BMD throughout the recovery period with versus without ORIF.

Our study has several limitations. We assessed radiographic and not clinical outcomes, including limb mobility during and after treatment, range of motion, grip strength, return to function, return to work/sports, therapy attendance, pain, or general amount of activity of the patient; these factors were unaccounted for and could have influenced our study outcomes. We only included patients from 2 clinical sites, limiting the generalizability of the results. Our sample sizes were smaller than expected because of many cases of missing, inaccessible, or inadequate numbers of radiographs. We could not differentiate the presence or absence of 6-week radiographs as a matter of routine or because of clinical factors. All these factors increase the risk of inadvertent selection bias. We only collected radiographic data for 2 time points, limiting further analysis of 2MCP/DO trends over time. History of osteopenia or osteoporosis, social history (smoking, alcohol, recreational drug use), DEXA results, and osteoporosis treatments were either missing or inconsistently documented in patient charts and were therefore not collected. We also do not know if preexisting osteopenia or osteoporosis influenced the development of DO in our cohorts. We did not measure BMD directly, using 2MCP as a validated proxy. How this reflects systemic BMD changes over short time intervals is unknown. However, it could potentially serve as a useful research tool in the study of bone loss during DRF recovery. Additionally, a better understanding of these changes in 2MCP as related to systemic BMD could impact how 2MCP is used in bone health evaluation and guiding treatment. Matching and extending the time points in our study with DEXA results would give valuable insight into local versus systemic changes in BMD following DRF. This could inform future recommendations on when and what type of BMD screening should occur and how results should be interpreted and addressed following DRF.
