# Supplementary material for: Development of Osteopenia During Distal Radius Fracture Recovery
Source: J Hand Surg Glob Online. 2022 Sep 27;4(6):315–9. doi: 10.1016/j.jhsg.2022.09.001 (PMC9678719; doi:10.1016/j.jhsg.2022.09.001)
Supplement: Appendix A [file mmc1.docx]

|  | OR (95% CI) | *p*-value |
| --- | --- | --- |
| Operative Status | | |
| Nonoperative | Ref |  |
| Operative | 0.59 (0.36 – 0.94) | **0.03** |
| Age | 1.02 (1.00 – 1.04) | **0.003** |
| Sex | | |
| Male | Ref |  |
| Female | 1.18 (0.69 – 2.04) | 0.53 |
| Race | | |
| Caucasian | Ref |  |
| Black | 1.07 (0.62 - 1.86) | 0.79 |
| Other | 0.83 (0.28 – 2.49) | 0.74 |
| Unknown | 0.96 (0.32 – 2.92) | 0.94 |
| LaFontaine Criteria | | |
| Stable | Ref |  |
| Unstable | 1.23 (0.78 – 1.94) | 0.38 |
| AO/OTA Classification | | |
| A1 | Ref |  |
| A2 | 1.84 (0.21 – 15.77) | 0.58 |
| A3 | 1.34 (0.15 – 12.25) | 0.80 |
| B1 | 2.07 (0.23 – 18.38) | 0.51 |
| B2 | 1.56 (0.11 – 20.85) | 0.74 |
| B3 | -- | -- |
| C1 | 1.59 (0.18 – 13.79) | 0.67 |
| C2 | 1.33 (0.15 – 11.60) | 0.80 |
| C3 | 1.38 (0.16 – 12.12) | 0.77 |
| CCI Category | | |
| Low comorbidity | Ref |  |
| Medium Comorbidity | 1.62 (0.85 – 3.08) | 0.14 |
| High Comorbidity | 1.86 (0.98 – 3.56) | 0.06 |
| BMI | | |
| Underweight | Ref |  |
| Normal | 0.96 (0.31 – 3.03) | 0.95 |
| Overweight | 1.07 (0.34 – 3.38) | 0.91 |
| Obese | 1.11 (0.35 – 3.51) | 0.86 |
| Bolded values are statistically significant.  DO = disuse osteopenia; OR = odds ratio; Ref = reference; AO/OTA = AO Foundation/Orthopaedic Trauma Association; CCI = Charlson Comorbidity Index; BMI = body mass index; 95% CI = 95% confidence interval. | | |

**Appendix A. Bivariate Analysis of Operative Status, Patient and Fracture Characteristics with Odds of Developing DO**
