# Supplementary material for: Development of Osteopenia During Distal Radius Fracture Recovery
Source: J Hand Surg Glob Online. 2022 Sep 27;4(6):315–9. doi: 10.1016/j.jhsg.2022.09.001 (PMC9678719; doi:10.1016/j.jhsg.2022.09.001)
Supplement: Appendix B [file mmc2.docx]

|  | N (%) or Mean (SD) | OR (95% CI) | *p*-value |
| --- | --- | --- | --- |
| Abnormal Parameters on Injury Radiographs | | | |
| Volar Tilt >10° dorsal | 369 (73.36) | 0.63 (0.38 – 1.03) | 0.06 |
| Radial Inclination <18 or >28° | 228 (45.51) | 1.12 (0.71 – 1.77) | 0.63 |
| Radial Height <10 mm | 205 (41.00) | 0.80 (0.50 – 1.28) | 0.35 |
| Ulnar Variance <0 or >2mm | 292 (58.40) | 1.08 (0.68 – 1.73) | 0.73 |
| Intra-articular Step-Off >1mm | 232 (45.85) | 0.80 (0.50 – 1.26) | 0.33 |
| Change in Radiographic Parameters | | | |
| Volar Tilt | -0.63 (8.84) | 1.00 (0.97 – 1.02) | 0.53 |
| Radial Inclination | -0.26 (5.01) | 1.00 (0.95 – 1.04) | 0.94 |
| Radial Height | -0.34 (2.71) | 1.01 (0.93 – 1.09) | 0.84 |
| Ulnar Variance | 1.10 (2.18) | 0.98 (0.88 – 1.09) | 0.70 |
| Intra-articular Step-Off | 0.01 (0.67) | 1.24 (0.89 – 1.73) | 0.19 |
| DO = disuse osteopenia; OR = odds ratio, SD = standard deviation, 95% CI = 95% confidence interval. | | | |

**Appendix B. Bivariate Analysis of Abnormal and Change in Radiographic Parameters with Odds of Developing DO (N=93)**
