# Supplementary material for: Development of Osteopenia During Distal Radius Fracture Recovery
Source: J Hand Surg Glob Online. 2022 Sep 27;4(6):315–9. doi: 10.1016/j.jhsg.2022.09.001 (PMC9678719; doi:10.1016/j.jhsg.2022.09.001)
Supplement: Appendix C [file mmc3.docx]

| **Variable** | **VIF** | **Tolerance** |
| --- | --- | --- |
| Age | 2.83 | 0.353 |
| Operative Status | 1.03 | 0.971 |
| CCI Category | 2.83 | 0.354 |
| Mean VIF: 2.23 | | |
| VIF = variance inflation factor; CCI = Charlson Comorbidity Index. | | |

**Appendix C. Statistics Evaluating for Multicollinearity**
